# A truncated form of the Carbon catabolite repressor 1 increases cellulase production in *Trichoderma reesei*

**DOI:** 10.1186/s13068-014-0129-3

**Published:** 2014-09-11

**Authors:** Thiago M Mello-de-Sousa, Rita Gorsche, Alice Rassinger, Marcio J Poças-Fonseca, Robert L Mach, Astrid R Mach-Aigner

**Affiliations:** Department for Biotechnology and Microbiology, Institute of Chemical Engineering, Vienna University of Technology, Gumpendorfer Str. 1a, A-1060 Wien, Austria; Department of Genetics and Morphology, Institute of Biological Sciences, University of Brasília, Campus Universitário Darcy Ribeiro, 70910-900 Brasília, DF Brazil

**Keywords:** *Trichoderma reesei*, *Hypocrea jecorina*, Rut-C30, Cellulases, Carbon catabolite repressor 1, Chromatin

## Abstract

**Background:**

Rut-C30 is a cellulase-hyperproducing *Trichoderma reesei* strain and, consequently, became the ancestor of most industry strains used in the production of plant cell wall-degrading enzymes, in particular cellulases. Due to three rounds of undirected mutagenesis its genetic background differs from the wild-type QM6a in many ways, of which two are the lack of a 83 kb large sequence in scaffold 15 and the partial lack of the gene encoding the Carbon catabolite repressor 1 (CREI). However, it is still unclear, what exactly enhances cellulase production in Rut-C30.

**Results:**

The investigation of the expression of two genes encoding cellulases (*cbh1* and *cbh2*) and the gene encoding their main transactivator (*xyr1*) revealed that the presence of the truncated form of CREI (CREI-96) contributes more to the Rut-C30 phenotype than a general loss of CREI-mediated carbon catabolite repression (*cre1* deletion strain) or the deletion of 29 genes encoded in the scaffold 15 (83 kb deletion strain). We found that the remaining *cre1* in Rut-C30 (*cre1-96*) is transcribed into mRNA, that its putative gene product (Cre1-96) is still able to bind DNA, and that the CREI-binding sites in the upstream regulatory regions of the chosen CREI-target genes are still protected in Rut-C30. As it was previously reported that CREI acts on the nucleosome positioning, we also analyzed chromatin accessibility of the core promoters of CREI-target genes and found them open even on D-glucose in the presence of CREI-96.

**Conclusions:**

The lack of the full version of CREI in Rut-C30 corresponds with a partial release from carbon catabolite repression but is not completely explained by the lack of CREI. In contrast, the truncated CREI-96 of Rut-C30 exerts a positive regulatory influence on the expression of target genes. Mechanistically this might be explained at least partially by a CREI-96-mediated opening of chromatin.

## Background

The filamentous ascomycete *Trichoderma reesei* is a saprophyte known for its ability to efficiently degrade biomass material by plant cell wall (PCW)-degrading enzymes. A genome-wide analysis identified 10 cellulolytic and 16 hemicellulolytic enzyme-encoding genes in *T. reesei* [1], of which the two most prominent cellulose-degrading enzymes are the cellobiohydrolases CBHI and CBHII (EC 3.2.1.91) [2]. However, the defining feature of this fungus is the exceedingly high amount of secreted enzymes that provoked its industrial exploitation for their production. Next to the pulp and paper industry [3], the food and feed industry [4], and the textile industry [5], these enzymes are applied in the production of biofuels [4]. With regards to cellulosic ethanol, the production costs of the needed enzymes massively influence the price and the competitiveness of the end-product. As a result their efficient expression remains an important topic of research.

The ancestor of most current industry strains is Rut-C30 [6,7]. It was derived from the *T. reesei* wild-type isolate QM6a through three rounds of mutagenesis (ultraviolet (UV) light and N-nitroguanidine) followed by a screening for the release from carbon catabolite repression (CCR) and high cellulase activity [8–10]. Since then the study of the specific physiological and genetic changes in Rut-C30 has been of interest [7,11] and a number of properties have been identified, for example, a 83 kb large region located in scaffold 15, which encodes 29 genes is lacking in Rut-C30 [12,13]. Another important property of Rut-C30 is the lack of the full version of the Carbon catabolite repressor 1 (CREI, [14]) having left a *cre1* sequence that would only encode for one of the two zinc finger regions of CREI (96 aa long) [15]. The native CREI is a C_2_H_2_-type zinc finger protein with the consensus sequence 5′-SYGGRG-3′ [14], and is orthologous to CreA from *Aspergillus* sp. [16–18] and MIG1 from *Saccharomyces cerevisiae* [19].

In *T. reesei* CREI is known to act on a number of regulatory levels. Firstly, it directly represses transcription of several genes encoding for PCW-degrading enzymes, for example the *cbh1* gene [20], by binding to tandem and inverted repeats in their upstream regulatory regions (URRs) [20,21]. Secondly, in the presence of D-glucose it represses the expression of the main transactivator of PCW-degrading enzyme expression, the Xylanase regulator 1 (XYRI) [22,23]. XYRI is a Gal4-like Zn2Cys6 binuclear cluster protein, of which the expression can be induced by sophorose [6] or as above-mentioned, repressed by D-glucose. Notably, the extent of induction of *xyr1* gene expression directly correlates with the induction of *cbh1* and *cbh2* gene expression, which is not the case for other genes in the XYRI regulon [6]. As a third regulatory level, CREI has been reported to play an essential role in correct nucleosome positioning, for example in the promoters of the *cbh1* and *cbh2* genes [24,25]. Altogether, CREI plays a major role in the regulation of 250 genes, both in a repressing and inducing way [26].

In this study we investigated to which extent the two mentioned striking genetic properties of Rut-C30, namely the lack of the 83 kb in scaffold 15 and the absence of a full CREI contribute to its cellulase hyper-producing phenotype. A special focus was studying the role of the remaining truncated version of CREI with regard to the transcriptional regulation of target gene expression, the ability of DNA binding, and the influence on the chromatin structure. The PCW-degrading enzyme-encoding genes *cbh1* and *cbh2*, as well as *xyr1* as the gene encoding their transactivator, were chosen as the CREI-target genes to be analyzed.

## Results

### The expression profile of *cbh1*, *cbh2*, and *xyr1* in QM6a-CREI_96_ is closest to Rut-C30

**Figure 1:**
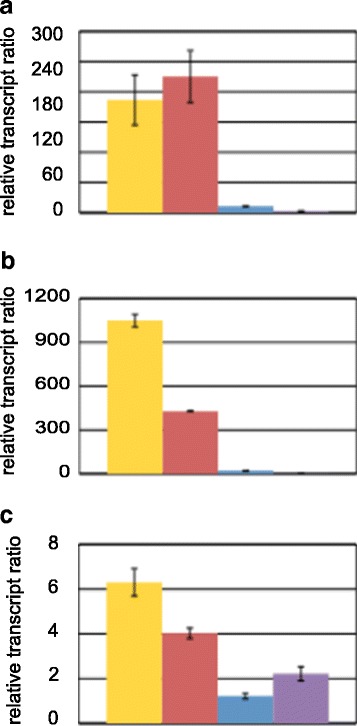
**Transcript analysis in** ***Trichoderma reesei*** **strains bearing Rut-C30-related mutations.** Rut-C30 (yellow bars), QM6a-CREI_96_ (red bars), the Δ*cre1*-strain (blue bars), and the Δscaff15-strain (purple bars) were pre-grown on glycerol and then transferred to medium supplemented with sophorose and incubated for 3 hours. The transcript level analysis of the *cbh1* **(a)**, *cbh2* **(b)**, and *xyr1* **(c)** genes was performed by qPCR using *sar1* and *act* genes for data normalization. Levels refer to the wild-type strain QM6a incubated without carbon source. The values are means from measurements and biological experiments (cultivations) performed in triplicate. Error bars indicate standard deviations.

In order to find out which genetic properties of Rut-C30 contribute to its cellulase hyper-producing phenotype we compared its expression profile to three transgenic strains. Two of them bear Rut-C30-related mutations, namely Δscaff15, which is a QM6a-derived strain with an 83 kb deletion in scaffold 15, and QM6a-CREI_96_, which bears the truncated CREI version like Rut-C30 does. We also included a QM6a-derived *cre1* deletion strain because the phenotype of Rut-C30 is sometimes associated with the lack of CREI. We investigated expression levels of *cbh1, cbh2,* and *xyr1* under sophorose-inducing conditions. Interestingly, the transcript levels of all three genes were most similar in QM6a-CREI_96_ compared to Rut-C30, while in the case of the other two strains (Δ*cre1* and Δscaff15) considerably lower levels were detected (Figure 1a, b, c). This result raised the consideration that regulatory molecular mechanisms are related to the truncated version of *cre1* remaining in the Rut-C30 genome.

### CREI sites in upstream regulatory regions of target genes are strongly protected from DNA methylation in Rut-C30

**Figure 2:**
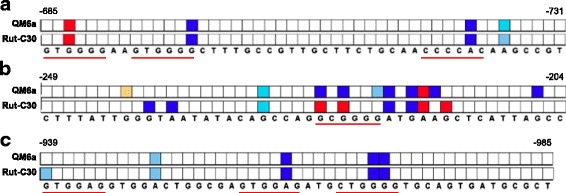
***In vivo*** **and** ***in vitro*** **footprinting analyses of URRs of CREI-target genes.** The *Trichoderma reesei* wild-type strain QM6a and Rut-C30 were pre-grown on glycerol and then incubated on D-glucose for 3 hours followed by DMS-induced *in vivo* methylation. An URR bearing functional CREI sites (underlined in red) of the *cbh1* **(a)**, *cbh2* **(b)**, and *xyr1* **(c)** genes was investigated, and methylated, naked DNA was used as the reference. Numbers indicate the position of the base upstream from ATG. Analysis of data and visualization was performed using ivFAST (*in vivo* footprinting analysis software tool) [27]. Only signals that are statistically different are considered. Protected bases are highlighted in red shades and hypersensitive bases are highlighted in blue shades. The three colour intensities each correspond to stronger differences between compared conditions; increasing colour intensity means more than 1.4-, 1.6-, and 1.8-fold difference in *cbh1* and *cbh2* **(a, b)**, and more than 2.4-, 2.6-, and 2.8-fold difference in *xyr1* **(c)**.

**Figure 3:**
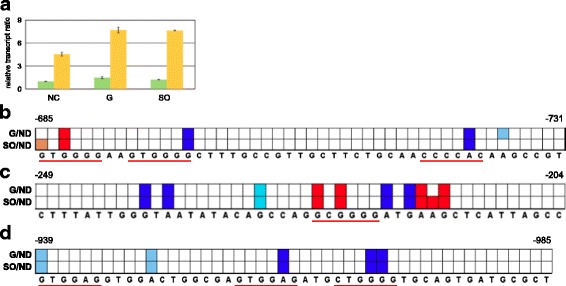
**Analysis of the abundance of CREI-96 in Rut-C30. (a)** The *Trichoderma reesei* wild-type strain QM6a (green bars) and Rut-C30 (yellow bars) were pre-grown on glycerol and then transferred to media supplemented with D-glucose (G), sophorose (SO) or without carbon source (NC), respectively, and incubated for 3 hours. The transcript level analysis of *cre1-96* was performed by qPCR using *sar1* and *act* genes for data normalization. Levels refer to the wild-type strain incubated without carbon source. The values are means from measurements in triplicates and three biological experiments (cultivations). Error bars indicate standard deviations. **(b-d)** *In vivo* and *in vitro* footprinting analysis of URRs of CREI-target genes in Rut-C30, which was pre-grown on glycerol and then incubated on D-glucose (G) or sophorose (SO) for 3 hours followed by DMS-induced *in vivo* methylation. An URR bearing functional CREI sites (underlined in red) of the *cbh1* **(b)**, *cbh2* **(c)**, and *xyr1* **(d)** genes each was investigated, and methylated, naked DNA (ND) was used as the reference. Numbers indicate the position of the base upstream from ATG. Analysis of data and visualization was performed using ivFAST [27]. Colour codes are the same as in Figure 2.

All above-mentioned analyzed genes (*cbh1*, *cbh2*, and *xyr1*) are under the regulatory influence of CREI [20,22,25]. To learn if the protection patterns against DNA methylation of the URRs bearing CREI sites of these genes differ between the wild-type QM6a and Rut-C30, we performed *in vivo*/*in vitro* footprinting analyses. URRs of all three genes were investigated after both strains were pre-grown on glycerol and then incubated on D-glucose for 3 hours followed by dimethyl sulphate (DMS)-induced *in vivo* methylation. The footprinting pattern obtained for all genes showed the same or even stronger DNA occupancy in Rut-C30 compared to QM6a (Figure 2a, b, c). This finding prompted us to analyze if the truncated *cre1* (*cre1-96*) in Rut-C30 is transcribed. Thus, after pre-growth both strains were transferred to media containing D-glucose as a repressing condition, sophorose as an inducing condition, and no carbon source as a control condition respectively, and incubated for 3 hours. While we detected a low basal transcript level (originating from the native *cre1* gene) in the wild-type, we found increased levels in Rut-C30, whereupon the increase was more pronounced on D-glucose and sophorose than without carbon source (Figure 3a). Since the *cre1-96* mRNA could be detected in high amounts in Rut-C30 regardless of the applied condition (D-glucose or sophorose), we again performed *in vivo/in vitro* footprinting to investigate if the DNA protection pattern changes condition-dependent. We analyzed the same URRs of *cbh1*, *cbh2,* and *xyr1* genes comparing the application of D-glucose and sophorose. As can be inferred from Figure 3b, c, d no condition-specific differences could be detected even though strong DNA occupancy at the CREI sites was observed. This fits the expression results and suggests that CREI-96 may no longer act in a D-glucose specific manner.

### The truncated CREI-96 protein of Rut-C30 can bind to DNA *in vitro*

**Figure 4:**
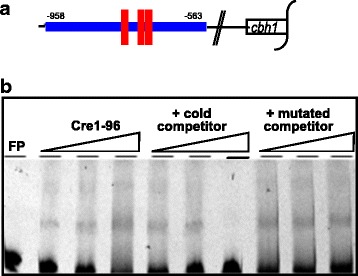
**Analysis of the DNA-binding ability of the CREI-96 protein. (a)** The EMSA was performed with a fluorescently labelled *cbh1* URR fragment (4 ng) bearing three functional CREI-sites (red bars). **(b)** Applied were increasing amounts of the heterologously expressed and thrombin-cleaved CREI-96 protein (500, 1000, and 2000 nM), and 2000 nM of CREI-96 together with increasing amounts (50-fold, 100-fold, and 200-fold) of a cold competitor (unlabeled probe), and 2000 nM CREI-96 together with increasing amounts (50-fold, 100-fold, and 200-fold) of a mutated cold competitor. FP, free probe.

The results described above raise the question of whether the putative CREI-96 protein is still able to bind to DNA, especially because it lacks one of the two zinc fingers. To answer this question, we performed an electrophoretic mobility shift assay (EMSA) using the URR of *cbh1* covering three functional CREI-binding sites as a probe (Figure 4a). The applied CREI-96 protein was heterologously expressed as GST-fusion protein and thrombin-cleaved before usage. The assay yielded two bands (Figure 4b), which represent most likely the binding of one or more CREI-96 proteins to the sites of the probe. Similar results were observed before reporting that shorter versions of CREI can still bind target DNA sequences yielding more than one band [28]. However, both complexes are specifically formed because the bands were diminished by adding a cold competitor, while they remained unchanged by adding a specifically mutated competitor (Figure 4b). Altogether, this supports the working hypothesis that the truncated CREI-96, which is putatively formed in Rut-C30, is still able to bind its DNA target sequences. It should be noted that in filamentous fungi other regulatory proteins are known that are also able to bind their DNA with a single zinc finger, such as the *Aspergillus nidulans* AreA [29].

### Higher gene expression corresponds with more open chromatin in QM6a-CREI_96_

**Figure 5:**
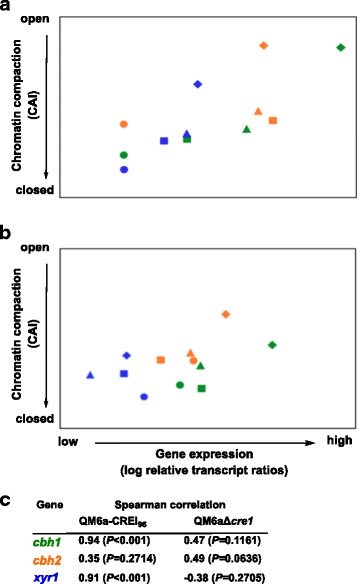
**Transcript and CHART analysis of** ***Trichoderma reesei*** **CREI-mutant strains during growth on D-glucose.** QM6a-CREI_96_ **(a)** and the Δ*cre1*-strain **(b)** were grown on D-glucose and samples were taken after 36 (dots), 39 (boxes), 42 (triangles), 45 (diamonds) hours. The *cbh1* (green), *cbh2* (orange), and *xyr1* (blue) genes were investigated. The gene expression analysis was performed by cDNA synthesis followed by qPCR, and transcript levels (log) are depicted on the x-axis. CHART-PCR was performed by Dnase I digestion followed by qPCR, and CAIs are depicted on the y-axis. In both cases *sar1* and *act* genes were used for data normalization and QM6a-CREI_96_ grown for 36 hours was the reference condition. All values are means from measurements in triplicates and three biological experiments (cultivations). Standard deviations were below 5%. Diagrams are identically scaled. **(c)** Spearman’s rank correlation coefficients were calculated (close to −1 indicates negative correlation; close to 0 indicates no linear correlation; close to 1 indicates positive correlation). The *P*-values for each correlation coefficient were calculated to determine significance of data (how different from zero). *P* >0.05 was considered as indicative of no linear correlation.

As we had strong indication that the truncated version of CREI-96 is present in Rut-C30 and still is able to bind the URRs of its target genes, we aimed to get more insights on its potential regulatory role. Consequently, QM6a-CREI_96_ and the *cre1* deletion strain were grown on D-glucose and samples were drawn after 36, 39, 42, and 45 hours of cultivation. Because CREI was reported to be involved in nucleosome positioning within the *cbh1* and the *cbh2* promoter of *T. reesei* [24,25], we investigated the chromatin packaging by applying chromatin accessibility real-time PCR (CHART-PCR) analysis of the core promoter regions of the *cbh1*, *cbh2*, and *xyr1* genes. Second, we investigated the expression of these genes by RT-qPCR to see if there was a correlation of transcript levels with the chromatin accessibility. We could detect a higher expression of *cbh1* and *cbh2* genes in QM6a-CREI_96_ compared to the Δ*cre1*-strain, which corresponded with a more open chromatin in the core promoters of the two genes in QM6a-CREI_96_ (Figure 5a, b). The higher transcript levels of cellulase-encoding genes in QM6a-CREI_96_ were reflected by increased enzyme activity (45.8 ± 1.5 U/mg dry weight;) measured after 45 hours of cultivation on D-glucose compared to the Δ*cre1*-strain (29.1 ± 1.8 U/mg dry weight). Interestingly, we found that in QM6a-CREI_96_ the gene expression increased with a simultaneous opening of chromatin, in particular in *xyr1* and *cbh1* (Figure 5a). On the other hand, we could not observe a correlation of gene expression and chromatin accessibility in the *cre1* deletion strain (Figure 5b).

**Figure 6:**
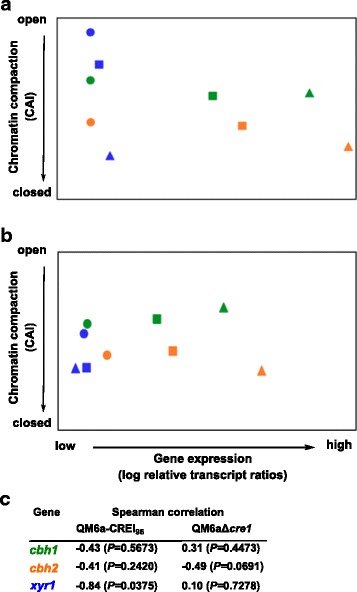
**Transcript and CHART analysis of** ***Trichoderma reesei*** **CREI-mutant strains during sophorose induction.** QM6a-CREI_96_ **(a)** and the Δ*cre1*-strain **(b)** were pre-grown on glycerol and thereafter incubated on sophorose for 30 (dots), 90 (boxes), and 180 (triangles) minutes. The *cbh1* (green), *cbh2* (orange), and *xyr1* (blue) genes were investigated. The gene expression analysis was performed by cDNA synthesis followed by qPCR, and transcript levels are depicted on the x-axis. CHART-PCR was performed by Dnase I digestion followed by qPCR, and CAIs are depicted on the y-axis. In both cases *sar1* and *act* genes were used for data normalization and QM6a-CREI_96_ incubated for 30 minutes was the reference condition. All values are means from measurements in triplicates and three biological experiments (cultivations). Standard deviations were below 5%. Diagrams are identically scaled. **(c)** Spearman’s rank correlation coefficients were calculated (close to −1 indicates negative correlation; close to 0 indicates no linear correlation; close to 1 indicates positive correlation). The *P*-values for each correlation coefficient were calculated to determine significance of data (how different from zero). *P* >0.05 was considered as indicative of no linear correlation.

To discover what this scenario looks like under inducing conditions, we applied the same experimental strategy on the two strains after pre-growth followed by incubation on sophorose for 30, 90, and 180 minutes. For *cbh1* and *cbh2* we observed in both strains an increase in transcript levels over time, which did not correlate in either of the two strains with a simultaneous opening of chromatin (Figure 6a, b). In the *xyr1* gene, transcript levels did not increase however, the chromatin became more compact over time (Figure 6a, b). Comparing the chromatin status of the two strains, the chromatin was slightly more accessible in QM6a-CREI_96._ (Figure 6a, b).

### CREI-96 upregulates the expression of a helicase-like transcription factor

**Figure 7:**
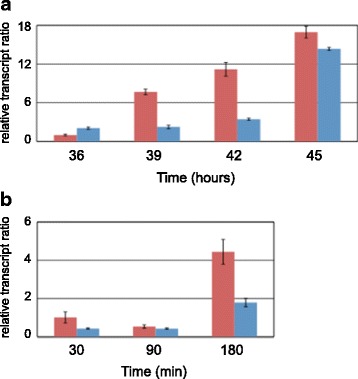
**Transcript analysis of** ***htf1*** **in** ***Trichoderma reesei*** **CREI-mutant strains.** QM6a-CREI_96_ (red bars) and the Δ*cre1*-strain (blue bars) were grown on D-glucose and samples were drawn after 36, 39, 42, and 45 hours **(a)** or pre-grown on glycerol and transferred to sophorose for 30, 90, and 180 minutes **(b)**. The transcript levels were analyzed by qPCR using *sar1* and *act* genes for data normalization and levels refer to QM6a-CREI_96_ grown for 36 hours or incubated for 30 minutes, respectively. The values are means from measurements in triplicates and three biological experiments (cultivations). Error bars indicate standard deviations.

Due to the results obtained from the CHART-PCR analyses, we assumed that the truncated CREI-96 was involved in chromatin remodelling in both a direct and indirect manner. Therefore, we searched the *T. reesei* genome for chromatin-remodelling proteins. We selected seven proteins, of which the annotation pointed to an involvement in chromatin rearrangement, for further investigation (Table 1). To determine a potential regulatory influence by CREI-96 we investigated their transcript levels in QM6a-CREI_96_ and the Δ*cre1*-strain. Amongst the seven genes we observed one with different transcript levels, namely the gene encoding a helicase-like transcription factor (Protein ID 44747). Here we detected strong upregulation in QM6a-CREI_96_ compared to the Δ*cre1*-strain, regardless of whether the samples from the D-glucose growth experiment (Figure 7a) or the sophorose replacement (Figure 7b) were investigated. We term the aforementioned gene in this study *htf1* (helicase-like transcription factor 1). It should be noted that in previous reports this gene was called *snf2*, although it is not homologous to SNF2 of *Saccharomyces cerevisiae* [30]. However, the increased expression of *htf1* in QM6a-CREI_96_ might be one reason for the observed change in chromatin.

**Table 1:** **Investigated candidate genes encoding ATP-dependent chromatin remodelling factors**

**Protein ID**	**Annotation** ^**a**^	**Provisory name**
44747	Helicase-like transcription factor HLTF/DNA helicase RAD5, DEAD-box superfamily	*htf1 (snf2)*
57935	Chromatin remodelling complex SWI/SNF, component SWI2 and related ATPases (DNA/RNA helicase superfamily)/ATPase	*snf2-like*
21557	Chromatin remodelling factor subunit and related transcription factors	*rsc8*
57608	Chromatin remodelling complex WSTF-ISWI, small subunit	*isw1*
109526	Chromatin remodelling complex WSTF-ISWI, small subunit	*isw2*
50539	SNF2 family DNA-dependent ATPase	*ino80*
58928	Chromodomain-helicase DNA-binding protein	*cdh1*

^a^according to TRIRE Joint Genome Institute - JGI - *Trichoderma reesei* v2.0 database.

## Discussion

For a long time its lack of the full version of CREI has been considered to be a positive genetic property of Rut-C30 because it leads to a partial carbon catabolite de-repression. However, Rut-C30 still possesses a short version of CREI, CREI-96. During this study it became clear that this protein still has a function and influences the expression of target genes, notably, in a positive manner. We found three ways in which CREI-96 mechanistically exerts its role as a regulatory protein. Firstly, it acts directly on promoters of target genes. The short CREI-96 (lacking one zinc finger) can still bind to target DNA sequences *in vitro*, which leads *in vivo* to a clear protection pattern from DNA methylation at CREI-binding sites (compare Figures 2 and 3). Obviously, contacting of CREI-binding sites in target genes by CREI-96 leads to a more open chromatin in the core promoter regions under repressing conditions (D-glucose). As expected, this effect was not observed in such a pronounced manner under inducing conditions (sophorose), as the core promoter there already has an open chromatin. If CREI-96 develops this de-regulating function by simply binding the DNA (possibly in high amounts) and thereby preventing nucleosome assembly, or if it is involved in a more complicated chromatin-DNA interaction mechanism, cannot yet be answered and this calls for further investigation.

Secondly, CREI-96 acts on the chromatin structure in an indirect way. The transcript levels of the *htf1* gene encoding a chromatin-remodelling protein are upregulated in the presence of CREI-96. Notably, in the wild-type strain QM6a bearing the native CREI we found *htf1* downregulated, specifically on D-glucose (data not shown). This is in good accordance with previous studies, in which this gene was reported to be repressed by CREI in the *T. reesei* strain QM9414 [26] and to be lowly expressed in QM6a on D-glucose [31]. It can be speculated that CREI-96 in this case reverses the antagonistic function of CREI on chromatin-remodelling proteins and even supports the opening of chromatin in an indirect way.

Thirdly, the loss of the auto-regulatory function of CREI is very likely. It should be considered that surprisingly high transcript levels of *cre1-96* were detected in Rut-C30 (Figure 3a). In *A. nidulans* it was observed that a low steady-state level of *creA* mRNA can be increased within minutes by adding a repressing carbon source. While a prolonged incubation with the repressing carbon sources then led to auto-repression, the incubation on a de-repressing carbon source maintained the high level [18]. In Rut-C30 the *cre1-96* transcript levels on D-glucose are as high as on sophorose, indicating a loss of the mentioned native auto-repression. This might also be the reason for the generally higher abundance of transcript in Rut-C30 compared to the wild-type strain QM6a which we observed during this study. An alternative or additional explanation for the generally higher abundance of *cre1-96* in Rut-C30 is mRNA stability. This may arise from less stability of the mRNA coding for the full-length protein, or a general increase in mRNA stability speculated to be caused by two mutations (protein ID 110423 and 66895) identified in the direct ancestor of Rut-C30 (NG14) [11]. However, it can be assumed that in Rut-C30 this results in high amounts of CREI-96 present in the cell, which is still able to bind DNA, thereby possibly changing the chromatin status in CREI-target genes.

Even if in previous studies truncated forms of CREI have been investigated, interestingly, none of them reported a positive effect of the shorter CREI version compared to a deletion of CREI. This is most likely due to a different experimental design applied. Nakari-Setälä *et al*. reported similar cellulase activities (volumetrically given) of strains bearing a deletion or a truncation of CREI when grown on lactose [32]. However, the investigated strains exhibited different growth behaviors (biomass formation and CO_2_-levels) [32]. In order to exclude any growth effects we studied a resting cell system under inducing conditions (sophorose). Such a highly standardized transfer experiment allows determination of the mechanistic influence of an isolated phenomenon (presence of CREI-96 or lack of CREI).

We also investigated growth conditions on D-glucose as this is certainly industrially relevant (enzyme production on high glucose-containing inducers). In this case we also observed differences in transcript levels between the two CREI mutant strains, which is in accordance with the results of a Northern analysis of *cbh1* on glucose by Nakari-Setälä *et al*. [32] and of the *cbh1* transcript level analysis by Ries *et al*. [24].

We noticed that the increased transcript levels in strain QM6a-CREI_96_ corresponded with an opening of chromatin on D-glucose, which was not found on sophorose. We propose that this different observation is likely due to the fact that the remodelling of chromatin usually becomes necessary under repressing conditions, while under inducing conditions certain regulatory factors and/or mechanisms have already ensured the open chromatin status. Obviously, the differences between the two CREI mutant strains observed on sophorose are primarily related to induction, chromatin opening, and expression of XYRI. During early-stage induction (30 minutes) the presence of sophorose leads to a rapid and higher chromatin accessibility of *xyr1* in QM6a-CREI_96_, which subsequently results in higher *xyr1* transcript levels (Figure 6). It was previously reported that the level of *xyr1* expression is directly linked to the expression of *cbh1* and *cbh2* [6]. Accordingly, we detected higher *cbh1* and *cbh2* transcript levels in the QM6a-CREI_96_ on sophorose.

## Conclusions

From the result obtained during this study we conclude that the truncated version of CREI present in Rut-C30 (CREI-96) should be considered as a discrete transcription factor with different properties than CREI. It acts in a direct manner on target URRs, but also contributes indirectly to a more open chromatin status by regulating a chromatin remodeler. Altogether it should be considered that Rut-C30 not only lacks CREI and the thereby mediated CCR, but also gains the now positively acting regulatory protein CREI-96.

Orthologs of CREI regulate CCR in numerous filamentous fungi including those used in biofuel production. Unfortunately, a simple deletion of *cre1* does not only lead to the desired release from CCR, but also to severe growth impairment. Consequently, molecular strain design can alternatively be based on the described CREI-96 truncation. This strategy does on one hand avoid growth deficiencies and the accompanying loss of productivity, and on the other hand additionally leads to a chromatin remodelling effect that results in increased expression of PCW-degrading enzymes.

## Materials and Methods

### Fungal strains

The following *T. reesei* strains were used throughout this study: the wild-type strain QM6a (ATCC 13631), Rut-C30, which was described as a high yielding cellulase mutant of QM6a (ATCC 56765) [10], a *cre1* deletion in QM6a (Δ*cre1*) [32], a 83 kb deletion corresponding to the large deletion in scaffold 15 of Rut-C30 in QM6aΔ*tmus53* (Δscaff15) [13,33], as well as a QM6aΔ*tmus53* strain bearing the truncated *cre1* of Rut-C30 (QM6a-CREI_96_) constructed during this study. All strains were maintained on malt extract agar or potato-dextrose-agar.

### Growth conditions

For carbon source replacement experiments mycelia were pre-cultured in 1 L Erlenmeyer flasks on a rotary shaker (180 rpm) at 30°C for 24 hours in 250 mL of Mandels-Andreotti (MA) medium [34] supplemented with 1% (w/v) glycerol as sole carbon source. A total of 10^9^ conidia per litre (final concentration) were used as inoculum. Pre-grown mycelia were washed and equal amounts were resuspended in 20 ml MA media containing 1% (w/v) D-glucose or 2 mM sophorose (Serva Electrophoresis, Heidelberg, Germany) as sole carbon source, or no carbon source respectively, and incubated for 30 minutes to 3 hours. For direct cultivation experiments the conidia were incubated in 250 mL Erlenmeyer flasks on a rotary shaker (180 rpm) at 30°C for 45 hours in 50 mL of MA medium supplemented with 1% (w/v) D-glucose as sole carbon source. Samples were derived from three biological replicates and were pooled before RNA extraction and chromatin digestion.

### Replacement of CREI in *T. reesei* QM6a

Transformation of *T. reesei* QM6aΔt*mus53* [33] was performed using two overlapping 3 kb-DNA fragments. The first fragment consisted of the truncated *cre1* of Rut-C30 amplified with primers RG186 and RG187 using genomic DNA as template and 3′-half of the expression cassette bearing the *Escherichia coli hph* marker gene amplified with primers RG188 and hph3′_fw using the plasmid pRLMex_30_ [35] as template. The second fragment consisted of the 5′-half of the expression cassette bearing the *E. coli hph* marker gene amplified with primers hph5′_rev and RG189 using pRLMex_30_ as template and the *cre1* 3′-flank from Rut-C30 amplified with primers RG190 and RG191 using genomic DNA as template. Protoplast transformation of QM6a was performed as described in United States patent number 8,323,931 using 2.5 μg of each DNA fragment in a co-transformation. Genomic integration of the full construct (*cre1-96*::*hph*) into the *cre1* locus was confirmed by southern blot analysis and DNA sequencing (Microsynth, Balgach, Switzerland).

### *In vivo* footprinting

*In vivo* methylation using DMS followed by ligation-mediated PCR was performed as described previously [27]. FAM (fluorescein amidite)-labelled fragments were analyzed by capillary gel electrophoresis (Microsynth) and results were analyzed using ivFAST [27].

### Analysis of transcript levels

Fungal mycelia were homogenized in 1 mL of peqGOLDTriFast DNA/RNA/protein purification system reagent (PEQLAB Biotechnologie, Erlangen, Germany) using a FastPrep(R)-24 cell disrupter (MP Biomedicals, Santa Ana, California, United States). RNA was isolated according to the manufacturer’s instructions, and the concentration was measured using the NanoDrop 1000 (Thermo Scientific, Waltham, Massachusetts, United States). Synthesis of cDNA from mRNA was carried out using the RevertAidTM H Minus First Strand cDNA Synthesis Kit (Thermo Scientific, Waltham, Massachusetts, United States) according to the manufacturer’s instructions. Quantitative PCRs were performed in a Rotor-Gene Q system (Qiagen, Hilden, Germany). All reactions were performed in triplicate. The amplification mixture (final volume 15 μL) contained 7.5 μL 2 × iQ SYBR Green Mix (Bio-Rad, Hercules, USA), 100 nM forward and reverse primer, and 2.5 μL cDNA (diluted 1:20). Primer sequences are provided in Table 2. Cycling conditions and control reactions were performed as described previously [36]. Data normalization using *sar1* and *act* as reference genes and calculations were performed as published previously [36].

**Table 2:** **Oligonucleotides used in this study**

**Name**	**Sequence (5′ - 3′)**	**Usage**
RG53	GAATTCAGATC	iv-FP, oligo-short
RG54	GCGGTGACCCGGGAGATCTGAATTC	iv-FP, oligo-long
RG89	[6-FAM]GTAGAGGCATGTTGTGAATCTGTGTCGGG	iv-FP, cbh1oligo3fw, EMSA
RG90	[6-FAM]GGTTGTATGCAAAACGCTCCGAGTCAGAC	iv-FP, cbh1oligo3rev, EMSA
RG215	CCAACGGCTT*GTGGGG*TTGCAGAAGCAACGGCAAAG*CCCCAC*TT*CCCCAC*GTTTGTTTCT^**a**^	EMSA
RG216	AGAAACAAAC*GTGGGG*AA*GTGGGG*CTTTGCCGTTGCTTCTGCAA*CCCCAC*AAGCCGTTGG	
RG221	CCAACGGCTT*GT* ***T*** *GGG*TTGCAGAAGCAACGGCAAAG*CCC* ***A*** *AC*TT*CCC* ***A*** *AC*GTTTGTTTCT^**b**^	
RG222	AGAAACAAAC*GT* ***T*** *GGG*AA*GT* ***T*** *GGG*CTTTGCCGTTGCTTCTGCAA*CCC* ***A*** *AC*AAGCCGTTGG	
RG178	TATCTCGAGTTTAGAAAAAAAAGCAGGT	pGEX-cre1-RG182 construction
RG182	ATTGGATCCATGCAACGAGCACAGTCTGCCGT	
RG186	TTGAGTGCAGACGTGTGTGTAATCTT	Construction of QM6a-CREI_96_
RG187	CCCTCCTTTGTTAGAAAAAAAAGCAGGTAATGG	
RG188	TTTTTTCTAACAAAGGAGGGAGACGAGGTTG	
RG189	CCTACATTGGATAACGGTGAGACTAGCGGCC	
RG190	TCACCGTTATCCAATGTAGGTAAGTAGTAAGGG	
RG191	GAATCAGTATTTTCTCATCTCCTTG	
hph3′_fw	GACCTGCCTGAAACCGAACTG	
hph5′_rev	GAAGAAGATGTTGGCGACCTCG	
actfw	TGAGAGCGGTGGTATCCACG	qPCR
actrev	GGTACCACCAGACATGACAATGTTG	
sar1fw	TGGATCGTCAACTGGTTCTACGA	
sar1rev	GCATGTGTAGCAACGTGGTCTTT	
cbh1f	GATGATGACTACGCCAACATGCTG	
cbh1r	ACGGCACCGGGTGTGG	
cbh2f	CTATGCCGGACAGTTTGTGGTG	
cbh2r	GTCAGGCTCAATAACCAGGAGG	
xyr1f	CCCATTCGGCGGAGGATCAG	
xyr1r	CGAATTCTATACAATGGGCACATGGG	
44747f	GCTCGAGCTGCAAGACAAGA	
44747r	GCGGAGATCCATGAGCTTCT	
epiactinTr_f	CTTCCCTCCTTTCCTCCCCCTCCAC	act CHART, region −226 to +24
epiactinTr_r	GCGACAGGTGCACGTACCCTCCATT	
episar1Tr_f	GTCAGGAAATGCCGCACAAGCAAGA	sar1 CHART, region −490 to −224
episar1Tr_r	TGTGTTTTACCGCCTTGGCCTTTGG	
epicbh1_2Tr_f	GGATCGAACACACTGCTGCCTTTAC	cbh1 CHART, region −301 to −27
epicbh1_2Tr_r	GGTTTCTGTGCCTCAAAAGATGGTG	
epicbh2_2Tr_f	TGCAGCGCAACACTACACGCAACAT	cbh2 CHART, region −355 to −62
epicbh2_2Tr_r	TGCGCCTCATACAGGGTCACAGTCC	
epixyr1_2Tr_f	CCGACAGCAGCAGTAGTCAGGTTTT	xyr1 CHART, region −216 to +35
epixyr1_2Tr_r	TAGGCAGAATAGCGACGGAGAGGAT	

^**a**^Italic letters indicate a CREI-binding site (5′-SYGGRG-3′).

^**b**^Bold letters indicate the introduced mutation in the CREI-binding site (5′-SYTGRG-3′).

### Plasmid construction

A 307 bp fragment was amplified from Rut-C30 genomic DNA using primers RG182 and RG178 and was inserted into the expression vector pGEX-4 T-2 (GE Healthcare Life Sciences, Little Chalfont, Buckinghamshire, United Kingdom) via *BamH*I/*Xho*I digestion yielding the plasmid pGEX-cre1-RG182 for heterologous expression of GST (glutathione S-transferase)-fused CREI-96.

### Protein expression and purification

*E. coli* BL21(DE3)pLysS (Promega, Madison, Wisconsin, United States) carrying pGEX-cre1-RG182 was cultivated in a 1 L Erlenmeyer flask on a rotary shaker (200 rpm) at 37°C in 300 mL LB medium supplemented with ampicillin (50 μg/mL) until an OD_600_ of 0.5 was reached. Protein expression was induced by adding IPTG to a final concentration of 0.5 mM followed by an incubation at 37°C for 3 hours. The cells were harvested by centrifugation and stored at −20°C overnight. GST-fusion protein (theoretical molecular weight 37 kDa) was purified from *E. coli* cell lysates using Glutathione-Superflow Resin (Qiagen, Hilden, Germany) according to the manufacturer’s instructions.

### Electrophoretic mobility shift assay (EMSA)

A 400-bp PCR product comprising the part of the 5′-URR region of *cbh1*, which contains 3 CREI-binding sites, was obtained with FAM-labelled primers RG89 and RG90 and used as probe (Table 2). The protein-DNA binding assay and non-denaturing polyacrylamide gel electrophoresis were performed essentially as previously described [37]. Binding was achieved by incubating increasing amounts of heterologously expressed, thrombin-cleaved CREI-96 (500 nM, 1000 nM, and 2000 nM) with 4 ng of the labelled, double-stranded DNA fragment in GST elution buffer (10 minutes at 22°C). Fluorescence and image analysis of the gels was carried out using a Typhoon 8600 variable mode imager (Amersham Bioscience, part of GE Healthcare, Little Chalfont, Buckinghamshire, United Kingdom). Competition experiments were performed using 2000 nM of protein together with increasing amounts (50-fold, 100-fold, and 200-fold) of either a cold competitor (an unlabeled double-stranded DNA probe obtained by annealing primers RG215 and RG216) or a mutated cold competitor (an unlabeled double-stranded DNA probe obtained by annealing primers RG221 and RG222). This probe bears in all three CREI-binding sites a mutation from 5′-SYGGRG-3′ to 5′-SYTGRG-3′, which was previously shown to prevent the binding of CREI *in vivo* and *in vitro* [21].

### Determination of cellulase activity

Cellulase activity in the culture supernatants was determined using AZCL HE-Cellulose (Megazyme International, Bray, Ireland) in 25 mM sodium acetate buffer pH 4.5 according to the manufacturer’s instructions. To measure biomass (dry weight), the cultures were harvested by filtration, washed with an equal volume of 0.8% NaCl solution, dried at 80°C for 24 hours, and weighed. Samples from two biological replicates and two technical replicates were measured.

### Chromatin accessibility real-time PCR (CHART-PCR)

DNase I digestion of chromatin and DNA extraction were carried out as described by Gonzalez and Scazzocchio [38] with minor modifications. Mycelia were harvested by filtration, pressed dry with filter paper, frozen in liquid nitrogen, and ground to a fine powder. Portions (100 mg) of the powder were suspended in 1 mL aliquots of nuclease digestion buffer (250 mM sucrose, 60 mM KCl, 15 mM NaCl, 0.05 mM CaCl_2_, 3 mM MgCl_2_, 0.5 mM DTT, and 15 mM Tris-HCl at pH 7.5), and 100-μL samples of the digestion mixture were incubated with 10 U of RQ1 RNase-free DNase I (Promega, Madison, Wisconsin, United States) for 2.5 minutes at 37°C. The reaction was stopped by adding 100 μL of 40 mM EDTA and 2% SDS, followed by two rounds of phenol-chloroform extraction and one round of chloroform extraction. Samples were then treated with 10 μg/mL of RNase A for 15 minutes at 37°C and precipitated with ethanol. DNA pellets were suspended in 100 μL of 5 mM Tris-HCl at pH 7.5. A control without DNase I was included to monitor endonuclease activity. qPCR analysis of the DNase I-treated samples was performed to measure the relative abundance of target regions. PCRs were performed in a Rotor-Gene Q system (Qiagen, Hilden, Germany). All reactions were performed in triplicate. The amplification mixture (final volume 20 μL) contained 10 μL 2 × iQ SYBR Green Mix (Bio-Rad, Hercules, USA), 200 nM forward and reverse primers and 10 ng of DNA. Primer sequences are provided in Table 2. Cycling conditions were as follows: 3 minutes initial denaturation at 95°C, followed by 40 cycles of 15 seconds at 95°C and 60 seconds at 60°C. The amount of intact input DNA of each sample was calculated by comparing the threshold values of the PCR amplification plots with a standard curve generated for each primer set using serial dilutions of genomic, undigested DNA. The chromatin accessibility index (CAI) was defined as:

1 $$ \mathrm{CAI}=1/\left(\mathrm{Ds}/\left(\left(\mathrm{Dc}1+\mathrm{Dc}2\right)/2\right)\right) $$

where Ds is the amount of intact DNA detected for each target region and Dc1 and Dc2 are the amounts of intact DNA detect for the promoter regions of *sar1* and *act* respectively, used as reference genes for normalization.

To access the relationship between the CAI and the transcript level, a two-tailed Spearman’s rank correlation coefficient was determined for each gene analyzed. The *P*-value for each correlation coefficient was calculated to determine the significance of the data.

### Selection of investigated genes encoding chromatin remodelling factors

Genes were selected by direct searching for SNF2, ISW1, ISW2, INO80, CDH1, RSC8 (the most prominent chromatin remodelling-related proteins characterized in yeast) in the *T. reesei* genome database [39]. Additional candidate genes were obtained by using BLASTp (basic local alignment search tool) search in the NCBI database employing respective *S. cerevisiae* sequences as baits to identify similar sequences in filamentous fungi. Subsequently, these sequences were used in a BLAST search in the *T. reesei* database.

## Abbreviations

BLAST: Basic local alignment search tool
CAI: Chromatin accessibility index
CHART-PCR: Chromatin accessibility real-time PCR
CREI: Carbon catabolite repressor 1
DMS: Dimethyl sulphate
EMSA: Electrophoretic mobility shift assay
GST: Glutathione S-transferase
ivFAST: *in vivo* footprinting analysis software tool
MA: Mandels-Andreotti
PCW: Plant cell wall
RT-qPCR: Reverse transcription quantitative PCR
URR: Upstream regulatory region
XYRI: Xylanase regulator 1
